# Run-Off Computed Tomography Angiography (CTA) for Discriminating the Underlying Causes of Intermittent Claudication

**DOI:** 10.1371/journal.pone.0152780

**Published:** 2016-04-07

**Authors:** Alexandra Preuß, Lars-Arne Schaafs, Thomas Werncke, Ingo G. Steffen, Bernd Hamm, Thomas Elgeti

**Affiliations:** 1 Department of Radiology, Charité-Universitätsmedizin, Berlin, Germany; 2 Institute for Radiology, Medizinische Hochschule Hannover, Hannover, Germany; University Hospital-Eppendorf, GERMANY

## Abstract

**Aim:**

To evaluate run-off computed tomography angiography (CTA) of abdominal aorta and lower extremities for detecting musculoskeletal pathologies and clinically relevant extravascular incidental findings in patients with intermittent claudication (IC) and suspected peripheral arterial disease (PAD). Does run-off CTA allow image-based therapeutic decision making by discriminating the causes of intermittent claudication in patients with suspected peripheral arterial disease PAD?

**Material and Methods:**

Retrospective re-evaluation of CTAs performed in patients with acute or chronic intermittent claudication (i.e., Fontaine stages I to IIB) between January 2005 and October 2013. Allocation to one of three categories of underlying causes of IC symptoms: vascular, musculoskeletal (MSK) or both. Clinically relevant extravascular incidental findings were evaluated. Medical records were reviewed to verify specific therapies as well as main and incidental findings.

**Results:**

While focused on vascular imaging, CTA image quality was sufficient for evaluation of the MSK system in all cases. The underlying cause of IC was diagnosed in run-off CTA as vascular, MSK and a combination in n = 138 (65%), n = 10 (4%), and n = 66 (31%) cases, respectively. Specific vascular or MSK therapy was recorded in n = 123 and n = 9 cases. In n = 82, no follow-up was possible. Clinically relevant extravascular incidental findings were detected in n = 65 patients (30%) with neoplasia, ascites and pleural effusion being the most common findings.

**Discussion:**

Run-off CTA allows identification of vascular, MSK, and combined causes of IC in patients with suspected PAD and can guide specific therapy. CTA also allowed confident detection of crEVIF although detection did not necessarily trigger workup or treatment.

## Introduction

Intermittent claudication (IC) is a typical symptom of peripheral arterial disease (PAD). In some cases, the patient’s history and clinical examination alone allow differentiation of PAD from other underlying conditions such as lumbar spinal stenosis: vascular claudication typically occurs after walking and is associated with a decreased ankle-brachial index (ABI) [1], whereas neurogenic claudication, e.g., caused by lumbar spinal stenosis, is typically associated with activity and spinal position [2]. However, due to differences in subjective perception of symptoms, the exact diagnosis often cannot be made on clinical examination alone and ultimately requires imaging [2–4].

Run-off computed tomography angiography (CTA) has become the method of choice for non-invasive imaging of the aorta and lower limb vessels in patients with suspected PAD [5] and is considered to be more accurate than arterial duplex sonography [6]. While still considered the diagnostic standard of reference for PAD, digital subtraction angiography (DSA) is currently being replaced by non-invasive imaging techniques such as CTA or magnetic resonance angiography (MRA) [7,8]. Several studies have shown that CTA is highly accurate in detecting arterial stenosis while avoiding common complications associated with invasive DSA [9]. Furthermore, CTA has become widely used because it is fast and well tolerated by patients and allows precise treatment planning even when using low-dose protocols [10,11].

Although the focus is on the vascular system, CTA allows assessment not only of the vascular situation of the aorta and lower extremities but also of the appearance of extravascular structures such as the musculoskeletal system [12,13]. To the best of our knowledge, a study investigating the incidence of nonvascular underlying causes and combined vascular and musculoskeletal pathologies in patients with intermittent claudication has not been done before.

The aim of the present study therefore was to evaluate run-off CTA of the abdominal aorta and lower extremities for identification of nonvascular underlying causes of IC and detection of clinically relevant extravascular incidental findings (crEVIF) in patients with intermittent claudication and suspected PAD. The study also aimed at determining to what extent run-off CTA could serve as an imaging method for guiding therapy by discriminating the origin of intermittent claudication in patients with suspected PAD.

## Material and Methods

### Patients

This retrospective study was approved by the institutional review board (IRB; EA4/058/13) of Charité-Universitätsmedizin Berlin. Written informed consent was given from all patients for using imaging data and medical records for study purposes and imaging data were not anonymized prior to analysis. The radiological database was screened for patients who underwent CTA because of acute or chronic intermittent claudication (i.e., symptoms corresponding to stages I to IIB according to Fontaine classification) between January 2005 and October 2013. Patients with symptoms indicating stage III or IV disease were excluded from our retrospective analysis since those stages are associated with stable rather than intermittent symptoms or always have a vascular pathology as the underlying cause. Categorization was based on clinical information.

### Imaging

Run-off CTA had a scan range extending from the costodiaphragmatic recess to the forefoot and was conducted either on a 64-slice CT scanner (Somatom Definition^®^, Siemens Healthcare, Erlangen, Germany) or on a 16-slice CT scanner (Somatom Sensation 16^®^, Siemens Healthcare, Erlangen, Germany). 64-slice CT was acquired with the following parameters: tube voltage 120 kVp, reference tube current-time product 120 mAs with tube current modulation CareDose4D^®^, rotation time 0.33 s; collimation 2 x 32 x 0.6 mm, pitch 0.75. CT scans on the 16-slice CT scanner were performed using the following acquisition parameters: tube voltage 120 kVp, reference tube current-time product 120 mAs with tube current modulation CareDose4D^®^, rotation time 0.75 s; collimation 16 x 1.5 mm, pitch 1.5.

CTA was performed with the patient supine. A dose of 100 ml of iomeprol 400 mg I/ml (Imeron 400^®^, Bracco, Milano, Italy), followed by a 60 ml saline flush, both at a flow rate of 4.0 ml/s, was injected using a dual-barrel injector (Stellant^®^, Medrad, Volkach, Germany) via a 20 G or larger IV cannula placed preferably in an antecubital vein. Arterial-phase images were obtained 10 s after bolus detection in the suprarenal aorta (threshold 250 HU, CareBolus^®^, Siemens Healthcare). For vascular assessment, images were first reconstructed using a soft kernel (B25f) with a field of view of 330 mm and an effective slice thickness of 1.0 mm (reconstruction interval 0.7 mm) for 64-slice CT and 2.0 mm (reconstruction interval 1.0 mm) for 16-slice CT. Second, images were reconstructed with a larger field of view adjusted to the patient’s size and using a slice thickness of 5.0 mm (64-slice CT) and 6.0 mm (16-slice CT) for display of the whole patient. Additionally, in every patient coronal and sagittal reformations as well as MIP reconstructions were done.

### Image review

CTA datasets were evaluated by two trained readers focusing on findings explaining symptoms of IC and clinically relevant extravascular incidental findings (crEVIF) requiring further diagnostic workup and/or immediate therapy according to the currently accepted state of the art [14–16]. All images were reviewed using Visage 7 (Pro Medicus Ltd., Richmond, Australia).

Patients were allocated to one of three categories based on the imaging finding best explaining symptoms of IC: 1 = vascular pathology (VASC), 2 = musculoskeletal (MSK) pathology or 3 = combined vascular and MSK pathology (COMB). Regarding MSK IC, we classified the following findings as possibly causing IC-like symptoms: absolute spinal stenosis (defined as a combination of bilateral facet arthrosis, disk bulging or herniation and hypertrophy of the ligamentum flavum causing narrowing of the sagittal diameter of the spinal canal), intervertebral disk herniation, vertebral body fracture, and severe joint diseases (coxarthrosis, gonarthrosis, chondrocalcinosis) [17–20].

Morphologic criteria were used to define crEVIF [15,16,21]. The following findings were considered as clinically relevant: lesions with imaging appearance suggesting malignancy or indirect signs of malignancy (e.g., double duct sign), infectious diseases with or without accompanying findings (e.g. effusion), hernia, organomegaly, injuries and urinary obstruction. Patients with crEVIF were assigned to subgroups of the aforementioned categories 1–3. To verify the major IC-related finding and incidental findings identified by CTA, we reviewed medical history forms and/or patient records. Following this review, patients were re-allocated to one of three groups based on what kind of therapy was initiated based on IC-related findings at CTA: 1 = therapy focused on detected vascular pathology, 2 = therapy focused on detected MSK pathology, 3 = no therapy noted in medical history. Treatment or follow-up of crEFIV was evaluated separately. Statistical analysis was performed using SPSS 23 for Macintosh OS X (IBM, Armonk, USA). Descriptive statistics of leading differential diagnosis for IC and incidental findings were performed. Means and standard deviations (SDs) of all acquired continuous variables were calculated.

## Results

Four hundred and twenty-nine patients with IC who had run-off CTA between January 2005 and October 2013 were identified. Of those, 214 patients had acute or chronic symptoms of intermittent claudication corresponding to Fontaine stages I—IIb. Mean age was 70 years (SD +/- 10.1 years). Sixty-five patients (29.4%) were women. All CTA datasets allowed assessment of the vascular tree and of the lumbar spine, hip and knee. No patients had to be excluded from retrospective analysis due to insufficient visualization of the spine, hip and knee.

**Fig 1:**
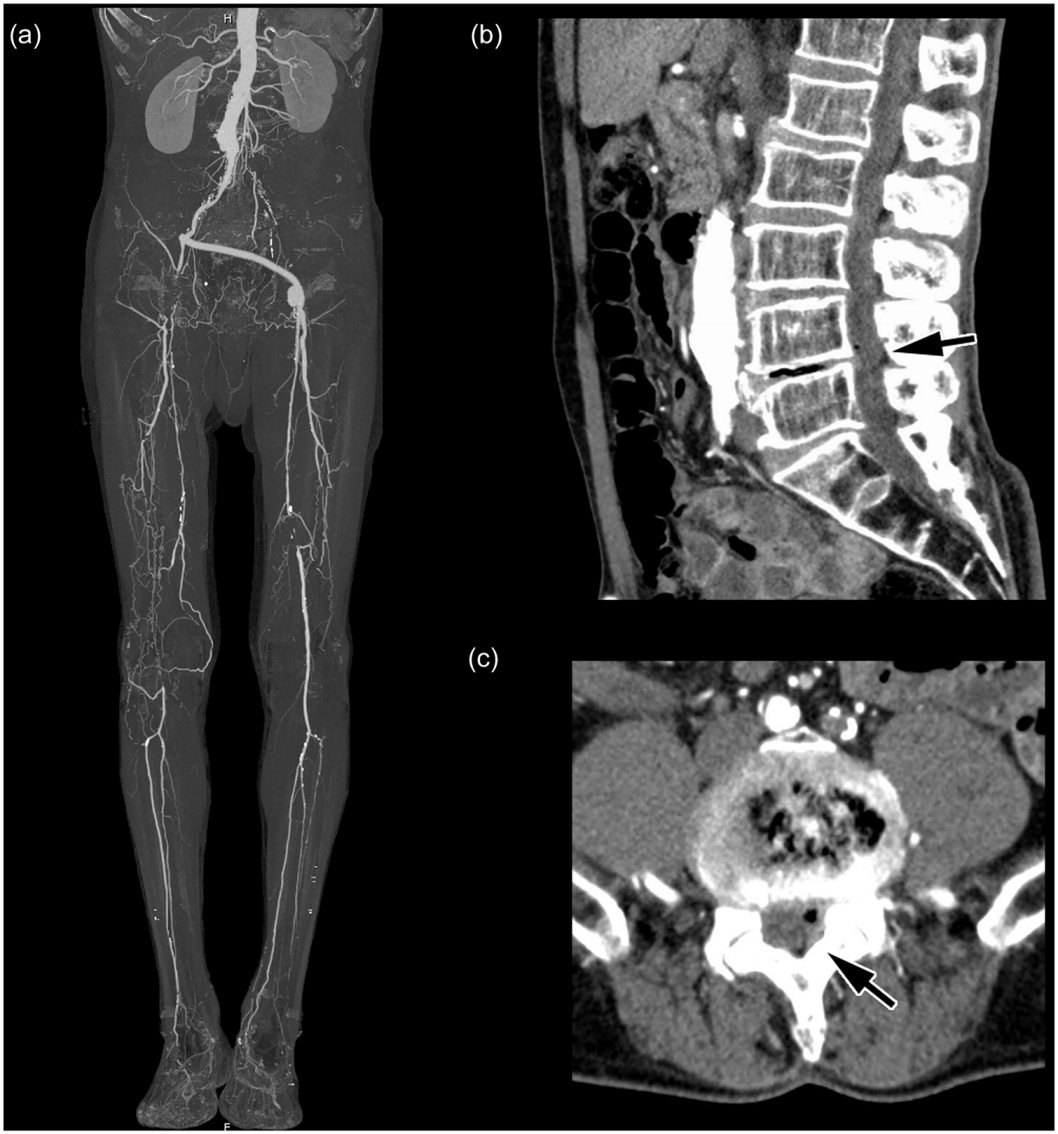
Example of a patient with known chronic PAD. The MIP (30° LAO) on the left shows the patient’s complex vascular situation with iliacofemoral crossover bypass and chronic SFA occlusions. The right side shows the lumbar spine with herniated vertebral disk between the fourth and fifth lumbar vertebrae and consecutive lumbar spinal stenosis (arrow).

**Fig 2:**
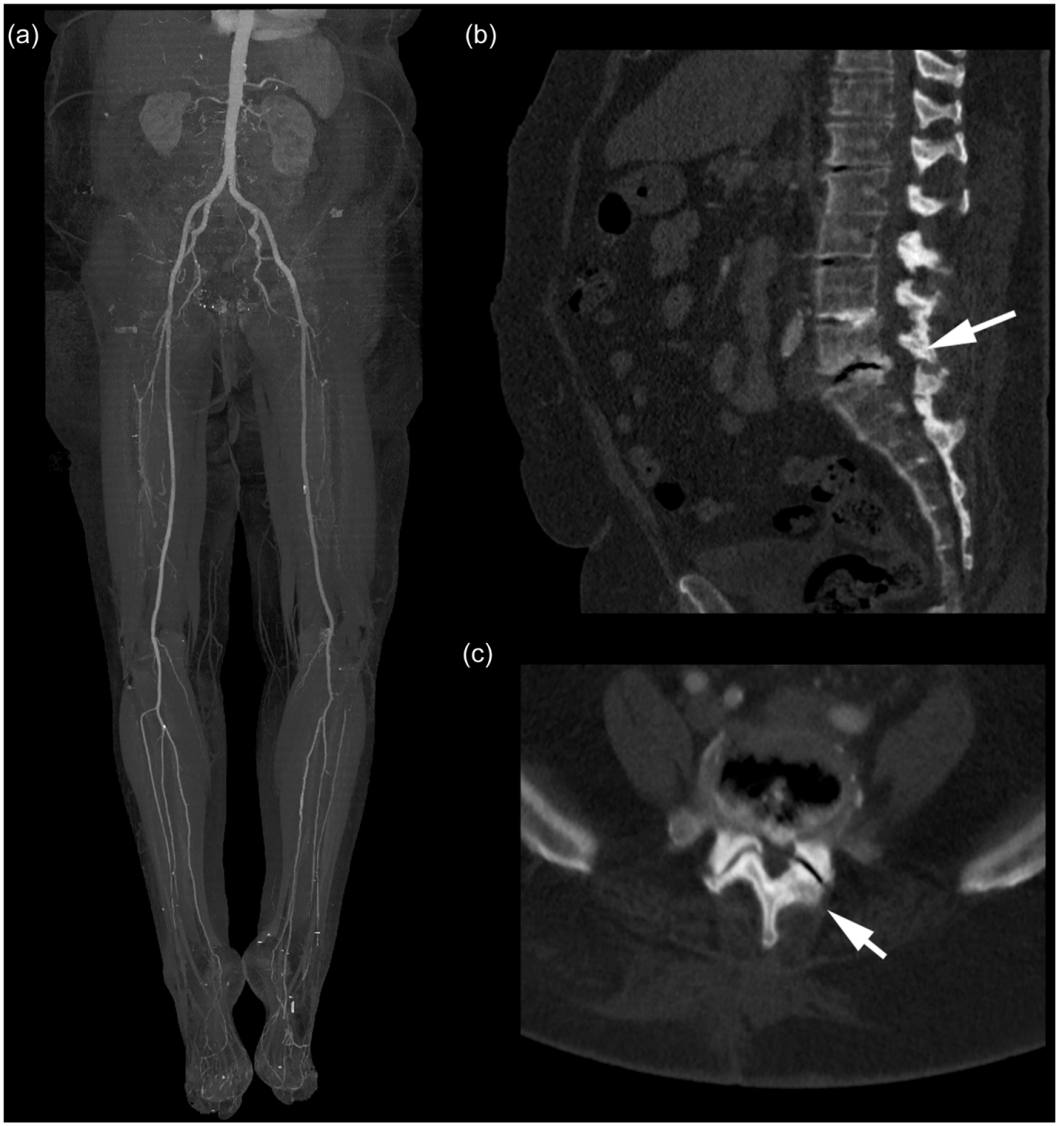
Example of a patient presenting with intermittent claudication. The maximum intensity projection (MIP, 30° left anterior oblique, LAO) presented on the left side clearly excludes relevant vascular pathology accounting for the clinical symptoms. On the right, a 4 mm average projection of the lumbar spinal canal clearly depicts a compression fracture of the fourth lumbar vertebra with spinal stenosis (arrow).

**Fig 3:**
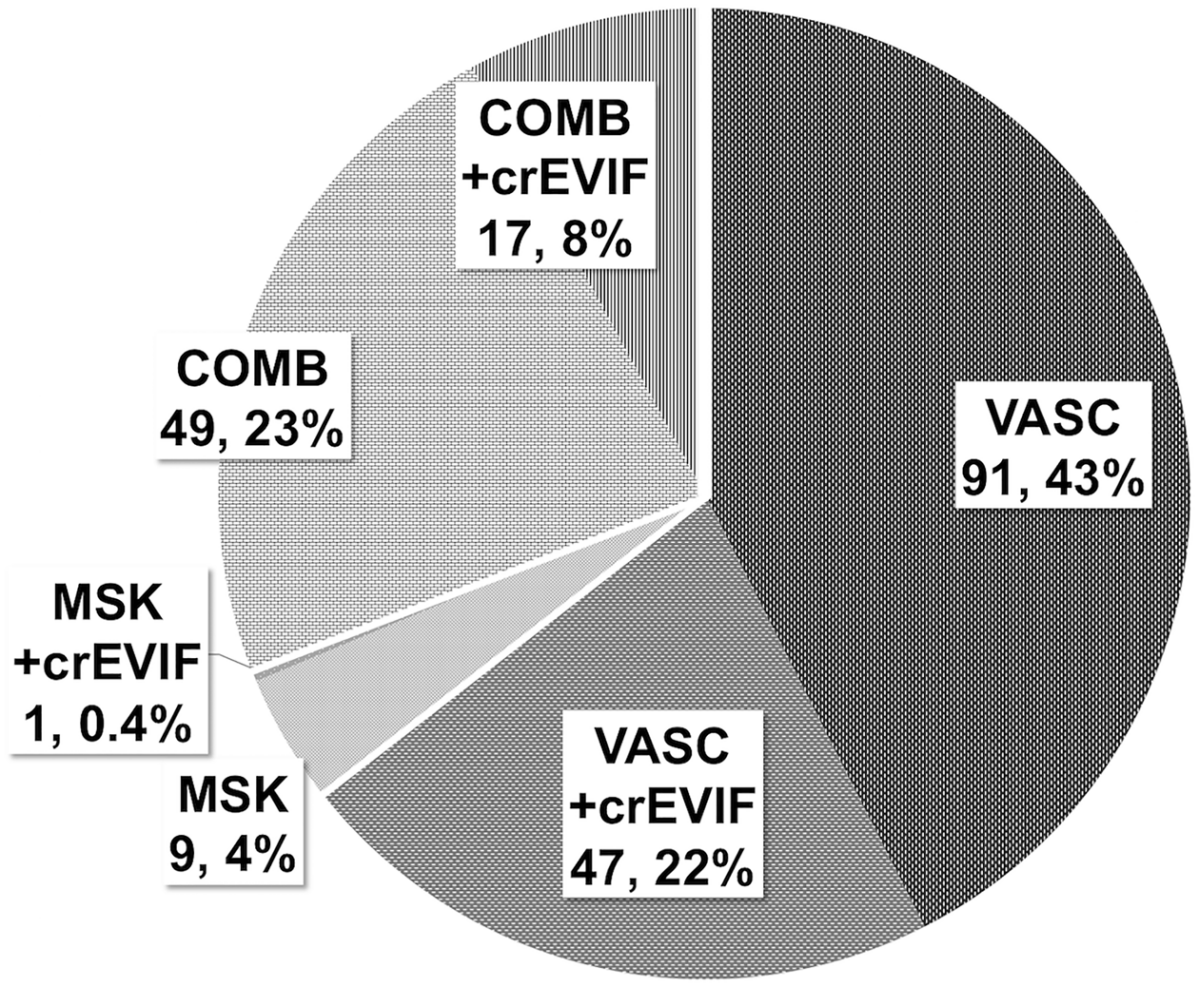
Pie chart for distribution of origin of intermittent claudication assessed with run-off CTA. The chart presents the distribution and incidence of vascular (VASC), musculoskeletal (MSK), and combined (COMB) causes of intermittent claudication. Additionally, the percentage of patients with clinically relevant extravascular incidental findings (crEVIFs) is displayed for each group (+crEVIFs, shaded area). In the vast majority of cases IC is due to vascular pathology (96%). In 31% of the cases coexisting musculoskeletal findings might also explain intermittent claudication. In only 4% of cases was MSK pathology identified as the only underlying cause.

An underlying vascular cause for symptoms of IC was detected by CTA in 138 cases (65%). In 66 cases (31%), vascular and MSK findings were identified, an example is presented in Fig 1. In the remaining 10 cases (4%), CTA demonstrated no vascular pathology but MSK findings explaining the patients’ symptoms, as shown in Fig 2. Clinically relevant EVIFs were found in 65 cases (30% of all patients). Patients with predominantly vascular pathologies had the highest percentage of crEVIF (47 of 138 cases; 22%), followed by patients with combined vascular and MSK pathologies (17 of 66 cases; 8%) and MSK pathologies alone (1 of 10; 0.4%). The distribution of underlying causes of IC and of subgroups with additional crEVIF is presented in Fig 3.

One hundred and twenty-three patients with vascular pathologies detected by CTA subsequently underwent vascular therapy (e.g., percutaneous transluminal angioplasty (PTA), surgically implanted vascular prosthesis), whereas 9 patients with MSK-related pathologies underwent orthopedic treatment (e.g., spinal fusion, spinal decompression). The data on treatment following CTA are summarized in Table 1. In 82 of 214 cases (38%), no therapy was mentioned in the medical records. Sixty-nine (84%) of those patients with no recorded therapy suffered from chronic IC-like symptoms rather than acute symptoms such as pain, paleness or pulselessness. Follow-up data on therapy or further diagnostic workup of detected crEVIF were available in 28 of 65 cases (43%). These data are summarized in Table 2.

**Table 1:** Distribution of therapies initiated after detection of either vascular or MSK pathologies by CTA.

Primary pathology detected in CTA	Therapy	Number of cases
Vascular (n = 123)	Embolectomy/ Surgical intervention	36
	PTA with stent placement	34
	PTA without stent placement	26
	Bypass	18
	Drug therapy	4
	Amputation	4
	Catheter-assisted lysis	1
Musculoskeletal (n = 9)	Spinal fusion	4
	Periradicular therapy	4
	Drug therapy	1

In both groups most patients had surgical or radiological interventions. Conservative therapy was only performed in a small fraction of patients. Numbers are given as absolute values.

**Table 2:** Distribution of crEVIFs.

crEVIF detected in CTA	Number of cases	Recorded therapy
Pleural effusion	9	Confirmation of cardiac insufficiency as the underlying cause leading to optimization of therapy (4)
		Pneumonia (2)
		No follow-up recorded (3)
Ascites	9	Reduction of ascites by drainage and/or drug therapy (3)
		No follow-up recorded (6)
Adrenal mass	7	Confirmed as primary or metastasis in follow-up CT (4)
		No follow-up recorded (3)
Double duct sign	7	No follow-up recorded (7)
Pulmonary mass	6	Confirmed as primary or metastasis in follow-up CT (4)
		No follow-up recorded (2)
Hernia	6	Surgical intervention for treating an inguinal hernia (1)
		No follow-up recorded (5)
Renal cyst (2F)	5	No follow-up recorded (5)
Urothelial carcinoma	3	Surgical intervention and/ or chemotherapy (3)
Pneumonia	2	Antibiosis (2)
Obstructive uropathy	2	No follow-up recorded (2)
Prostatitis	1	Intravenous antibiosis (1)
Renal cell carcinoma	1	Chemotherapy (1)
Ruptured spleen	1	Splenectomy (1)
Colitis	1	Confirmed as ischemic colitis with embolectomy performed as therapy of choice (1)
Sigmoid diverticulitis	1	Conservative treatment with intravenous antibiosis (1)
Hepatocellular carcinoma	1	No follow-up recorded (1)
Splenomegaly	1	No follow-up recorded (1)
Pancreatic mass	1	No follow-up recorded (1)
Hepatic lesion	1	No follow-up recorded (1)

Pleural effusion, ascites and direct or indirect signs of malignancy were the most common crEVIFs in our patient population. Medical records reported subsequent therapies or further examination in 28 cases.

## Discussion

Our retrospective analysis shows that, in an older patient population with intermittent claudication and suspected PAD, nearly a third of the patients have a combination of vascular and musculoskeletal findings explaining their symptoms. In 4% of the cases, musculoskeletal findings alone were identified as the underlying cause of IC. With the standard imaging protocol used for run-off CTA in this study not only assessment of the vascular tree but also identification of possible musculoskeletal causes of IC is possible. Therefore, run-off CTA enables stratification of IC patients for specific therapy.

To our knowledge, this is the first study to evaluate three categories, vascular, musculoskeletal and combined pathology, in patients with IC and the treatment initiatiated on the basis of CTA findings. Previous studies focused on the comparison of imaging methods—CT and magnetic resonance imaging (MRI)—in PAD or the diagnostic quality for detecting lumbar spinal stenosis in CT and MRI [18,22,23]. Musculoskeletal pathologies are the primary differential diagnosis in patients with suspected PAD, and our results show that run-off CTA allows adequate assessment of both pathologic conditions in one imaging procedure. In 31% of the cases, concomitant vascular pathology and clinically relevant musculoskeletal findings warranted a change and/or extension of vascular therapy. In 4% of the cases the therapeutic regimen was completely changed, as the suspected vascular pathology was not be confirmed by CTA. Since this retrospective analysis included patient population already suspected of having PAD, patients with lumbar spinal stenosis might be underrepresented and their percentage might be higher in a nonpreselected population.

As known from previous studies, the morphologic imaging appearance of osteoarthritic and intervertebral disk changes often does not correlate with disease severity or the patient’s clinical symptoms [24,25]. Specialized imaging methods using axial loading during MRI allow better identification of lumbar spinal stenosis but are not widely available[26]. The main limitation of this retrospective study is that a detailed clinical examination in the group of combined vascular and MSK pathologies after run-off CTA is missing, so that the leading cause of IC could not be fully elucidated. MSK findings might aggravate IC symptoms in part of the patients and should be recognized as a possible target relevant for further therapies.

Our further analysis revealed that 123 of 214 patients had vascular therapy based on imaging findings. In 9 cases specific musculoskeletal therapies were performed. Unfortunately, 82 cases (38%) were lost to follow-up, among them 69 patients with chronic IC. Only 13 patients had acute symptoms. Possibly these patients with chronic IC and well-known symptoms received conservative outpatient treatment with community doctors that was not recorded in the hospital database.

In this retrospective study, the images were reread by two trained readers in consensus focussing on MSK differential diagnosis and crEVIFs. In this way, possible underestimation of musculoskeletal findings or crEVIFs due to underreporting can be ruled out [14]. Of note, the results of the current study have changed our reporting regimen. Previously, a vascular radiologist alone read run-off CTAs. Now, double–reading with a general radiologist is the standard procedure. It was beyond the scope of our study to determine the rates of MSK and crEVIF findings identified in the the initial reading done by a vascular radiologist alone.

In addition to identification of the underlying causes of intermittent claudication, we detected clinically relevant extravascular incidental findings (crEVIFs) in 30% of patients, i.e. 65 cases. Newly diagnosed cancer was identified in 27 patients, which was confirmed on follow-up in 12 cases. Previously unknown infections were found in 5 cases. Both findings are highly important as cancer survival rates significantly decrease in patients with metastatic disease compared to local cancer [27]. In elderly patients, infectious diseases may remain clinically silent and might have a poorer clinical outcome [28]. The rate of newly detected or suspected malignancies (27 / 214 = 12.6%) is above the published range of 2.5% to 3.5%, which might be due to older age and the presence of known risk factors in patients with IC [15,29].

In 18 cases, previously unknown large pleural effusion or ascites led to immediate further clinical workup to differentiate between cardiac, hepatic, infectious, and neoplastic origin. In two cases, pleural effusion was caused by pneumonia. In 7 cases, optimization of specific cardiac insufficiency therapy was recorded. Although we do not have data on this it can be assumed that the radiological findings were reported to the transferring external physician and community doctor and led to initiation of specific therapy. The follow-up rate of the crEVIF was only slightly lower than described in the literature, i.e., 43%, compared to 58% or 61.7% [15,29,30]. However, the follow–up data of the 65 crEVIFs detected on run-off CTA confirmed that incidental detection led to specific treatment and presumably resulted in a more favorable clinical course for the patients. The incidence of crEVIFs differs greatly with the anatomic regions imaged, ranging between 6.7% in a cardiac CT study, 8.7% for the head and neck region and up to 48.8% in thoracoabdominal CT angiography [14,15,30,31]. Therefore, the rate of 30% in our study seems plausible. Of note, the patients of our cohort were significantly older and had concomitant vascular diseases (e.g., PAD or coronary artery disease) and other comorbidities such as renal impairment.

## Conclusion

Although the focus is on vascular assessment, run-off CT angiography of the aorta and lower extremities also allows differentiation of vascular and musculoskeletal causes of intermittent claudication in a single examination. It facilitates therapeutic decision-making since a considerable number of patients have clinically relevant musculoskeletal findings such as lumbar spinal stenosis or combined vascular and musculoskeletal pathology. Clinically relevant extravascular incidental findings are common on run-off CTA and can be identified on the basis of their CT morphology.
